# Hair cortisol concentrations correlate negatively with survival in a wild primate population

**DOI:** 10.1186/s12898-017-0140-1

**Published:** 2017-09-01

**Authors:** Josué H. Rakotoniaina, Peter M. Kappeler, Eva Kaesler, Anni M. Hämäläinen, Clemens Kirschbaum, Cornelia Kraus

**Affiliations:** 1 Department of Sociobiology/Anthropology, Georg-August University of Göttingen, Kellnerweg 6, 37077 Göttingen, Germany; 2 Behavioral Ecology and Sociobiology Unit, Deutsches Primatenzentrum, Kellnerweg 4, 37077 Göttingen, Germany; 3 Department of Biological Sciences, University of Alberta, Edmonton, AB T6G 2E9 Canada; 4 Department of Psychology, TU Dresden, Andreas-Schubert-Bau, Zellescher Weg 19, 01069 Dresden, Germany

**Keywords:** Cort-fitness hypothesis, Glucocorticoids, Stress, Fitness, Body condition, Parasitism, *Microcebus murinus*, Lemurs, Madagascar

## Abstract

**Background:**

Glucocorticoid hormones are known to play a key role in mediating a cascade of physiological responses to social and ecological stressors and can therefore influence animals’ behaviour and ultimately fitness. Yet, how glucocorticoid levels are associated with reproductive success or survival in a natural setting has received little empirical attention so far. Here, we examined links between survival and levels of glucocorticoid in a small, short-lived primate, the grey mouse lemur (*Microcebus murinus*), using for the first time an indicator of long-term stress load (hair cortisol concentration). Using a capture-mark-recapture modelling approach, we assessed the effect of stress on survival in a broad context (semi-annual rates), but also under a specific period of high energetic demands during the reproductive season. We further assessed the power of other commonly used health indicators (body condition and parasitism) in predicting survival outcomes relative to the effect of long-term stress.

**Results:**

We found that high levels of hair cortisol were associated with reduced survival probabilities both at the semi-annual scale and over the reproductive season. Additionally, very good body condition (measured as scaled mass index) was related to increased survival at the semi-annual scale, but not during the breeding season. In contrast, variation in parasitism failed to predict survival.

**Conclusion:**

Altogether, our results indicate that long-term increased glucocorticoid levels can be related to survival and hence population dynamics, and suggest differential strength of selection acting on glucocorticoids, body condition, and parasite infection.

**Electronic supplementary material:**

The online version of this article (doi:10.1186/s12898-017-0140-1) contains supplementary material, which is available to authorized users.

## Background

Identifying the links between physiological traits and fitness is vital for understanding the proximate mechanisms of selection that regulate natural populations. Glucocorticoid (GC) hormones are commonly employed as a biomarker of health or relative condition, both at the individual and the population level, since they mediate an array of physiological processes that can directly or indirectly impact fitness [1, 2]. As part of the hypothalamic-pituitary-adrenal (HPA) axis activity, GCs play a key role in the reallocation of resources in response to actual or perceived ecological challenges, such as inclement climatic conditions or predation pressure, that are associated with increased energetic needs [3–5]. Whereas the increase in GCs during acute stress is generally adaptive, chronic elevation of GC levels can compromise reproduction, immune function, and ultimately survival, thus reducing fitness [6–10]. For instance, individuals of various taxa have been found to potentially suffer fitness consequences of high GC concentrations through their increased susceptibility to parasite infection because of the immunosuppressive effects of GC [11–15]. The negative relationship between GC levels and fitness is at the core of the “Cort-fitness hypothesis”, which posits that high levels of baseline GCs indicate poor individual or population condition [16, 17].

However, to date, tests of the Cort-fitness hypothesis in wild populations are rare [9, 16], and in some of the few studies that attempted to do so, this hypothesis failed to receive empirical support [reviewed in 16]. Plausible explanations of this failure include the lack of repeatability of both the cort-fitness relationship and GC measures. Indeed, the cort-fitness relationship has been frequently described to be context-dependent, and factors such as sex and reproductive strategy may influence the nature of this correlation [18, 19]. Furthermore, GCs are known to show strong fluctuations over time [5, 20] and within-individual variability can potentially mask the effect of GC variation on fitness among individuals [20]. The majority of studies that have examined this correlation were based on a single sampling of GCs, using established biomarkers of acute stress (i.e., serum, saliva or urine), potentially biasing estimates of overall individual condition. Hence, when multiple sampling is not possible, the use of a biomarker of chronic stress might better illuminate this relationship.

Recently, the measurement of hair cortisol concentration (HCC) has emerged as a promising new tool for monitoring long‐term HPA axis activity [21–23]. Free circulating GCs are thought to be incorporated into the hair shaft throughout its growth [24, 25]. Therefore, HCC is unaffected by potential short-term fluctuation in cortisol secretion and allows an assessment of accumulated cortisol levels over a wider time window compared to traditionally used matrices. Moreover, cortisol levels in hair were proven to be highly stable and consistent within individuals [26–28]. While HCC has been applied to monitor individuals’ response to adverse conditions in several species (e.g., humans: [29]; chimpanzees: [30]; vervet monkeys: [31, 32]; marmosets: [33]; squirrels: [34]; cows: [35]; wolves: [36]), to our knowledge, no attempt was previously made to connect this indicator to measures of fitness in a wild population.

Studies of small, short-lived species can be advantageous when assessing the relationship between GCs and fitness in a wild setting, because in such study systems, physiological state can be measured repeatedly throughout the individual lifespan. The grey mouse lemur (*Microcebus murinus*), a small-bodied nocturnal primate (Lemuriformes: Cheirogaleidae), presents several key features allowing investigations of the potential relationship between individual condition and fitness. First, its average lifespan in the wild is 2–3 years, with a high annual turnover of around 50% [37, 38]. Second, in its highly seasonal habitat, strong fluctuation of water and food availability affects the feeding behaviour of *M. murinus* [39], but also several health indicators such as body mass, parasitism and levels of faecal GC metabolites [37, 40, 41]. Condition-dependent mortality is suggested to occur in mouse lemurs [37], and risky behaviour significantly influences male mortality during the breeding season [38]. While predation has been invoked as the probable leading cause of mortality [42], the proximate physiological mechanisms accompanying this non-random mortality remain unknown.

In this study, we test the hypothesis that high HCC (as a measure of long-term stress level) is related to individual survival in a wild population of *M. murinus*. Additionally, we assess the power of two other health indicators (size-adjusted body mass and patterns of parasitism) to predict survival. Specifically, we evaluate the potential link between these three health indicators and survival at two different levels. First, in order to define a general pattern, we test whether they can predict survival by assessing their relationship on a semi-annual basis, following the seasonal fluctuation of environmental conditions [43]. Second, we estimate the significance of individual condition under a specific context of high energetic demands by focusing on survival rates at the end of the dry season. The short mating season occurs during this period [44, 45], and it is associated with the lowest body mass and highest faecal GC metabolite levels [37, 40, 46]. We therefore predicted that individuals with high HCC suffer from lower survival. Furthermore, as individuals in poor health should be more vulnerable to ecologically adverse conditions, we expect survival to be positively correlated with general body condition, while individuals that exhibit a high degree of parasitism should face higher mortality.

## Methods

### Study site and population

The study was conducted in Kirindy Forest, which is a concession operated by the CNFEREF (Centre National de Formation, d´Etudes et de Recherche en Environnement et Foresterie), located approximately 60 km north of Morondava and a part of a dry deciduous forest in central western Madagascar [43]. We focused on a population of *M. murinus* from a 25 ha area (500 × 500 m) locally known as N5. This population has been continuously monitored since 2002.

### Capture-mark-recapture

As a part of the long-term live capturing protocol, we conducted monthly capture sessions during the mouse lemurs’ active period (from September to April) between 2012 and 2014. Trapping sessions consisted of three consecutive nights of capturing, using Sherman live traps baited with banana. Traps were set at 25 m intervals, at the intersections of a grid system of foot trails, in the late afternoon at 40–200 cm height, and checked in the early morning the next day. After being anesthetized with 0.02 ml ketamine (Ketavet^®^, Pfizer, Germany), captured animals were individually marked (or only identified without anesthetization if recaptured) with a subcutaneous transponder (Trovan EURO ID, Germany) and sexed, and standard field measurements, such as head width and body mass, were recorded. Hair samples for cortisol analysis and faecal samples for parasitology were also collected during capture sessions. While hair samples and morphometric measurements were collected semi-annually (in September–October and March–April), faecal samples were obtained opportunistically at a monthly rate.

To assess the relationship between HCC and semi-annual survival probabilities, we used the results of capture sessions held in October 2012, 2013, 2014, April 2013, and March 2014, during which a total of 171 individuals (74 females, 97 males) were captured. The same dataset, except the October 2014 session, was used to assess the effect of body condition on semi-annual survival probabilities, for a total of 149 individuals (63 females, 86 males). The link between survival probabilities and the health indicators (HCC, body condition, and parasitism) over the reproductive period was assessed by using data collected during monthly trapping sessions between September 2012 and April 2013. This dataset included 48 individuals (16 females and 32 males).

### Sample collection and analysis

#### Assessment of hair cortisol concentration

In order to avoid potential variation of hair cortisol concentration (HCC) from different body regions [47, 48], we collected hair samples consistently from the animals’ dorso-caudal region, where pelage coloration was reported to vary little across individuals and sexes [49], using a pet grooming clipper (Aesculap Isis GT 420). The detailed protocol for washing and extracting hair cortisol is described by Gao et al. [50], and all laboratory analyses were conducted at the University of Dresden (Germany). As a minor modification to the original protocol, because the hair structure of *M. murinus* prohibited the measurement of individual hair, we extracted cortisol using 7.5 mg of sample after washing (twice in 3 ml isopropanol for 3 min) and drying. The sample was further incubated with 40 μl internal standard and 2.4 ml methanol for 18 h at room temperature in a glass vial. After centrifugation at 10,000 rpm for 3 min, 1.6 ml of the clear supernatant was dried at 50 °C under a constant stream of nitrogen. The dry residue was re-suspended using 175 μl double-distilled water, 100 μl of which was used for cortisol concentration determination with liquid chromatography tandem mass spectrometry (LC–MS/MS). This assessment was performed using a Shimadzu HPLC-tandem mass spectrometry system (Shimadzu, Canby, Oregon) coupled to an ABSciex API 5000 Turbo-ion-spray triple quadrupole tandem mass spectrometer (AB Sciex, Foster City, California) with purification by on-line solid-phase extraction [50].

Although the mode (synchronous or asynchronous) and rate of hair growth are unknown for mouse lemurs, we are confident that the amount of hair we used for the HCC analysis is sufficient to accurately reflect a mean baseline cortisol concentration for this species. Indeed, as *M. murinus* has very dense fur, consisting of very thin hair. The number of hairs in a 7.5 mg sample (containing whole strands) is largely above 50–100 hairs, the number recommended by Fourie et al. [51] when studying medium-sized primates. Also, we observed that hair did not fully regrow after a month, motivating us to sample hair only at a semi-annual rate.

#### Assessment of general body condition

Instead of using body mass (BM) per se, we computed the scaled mass index (SMI) to assess body condition, thus controlling for the allometric relationship between body mass and body size [52]. This index yields an individual value of body mass standardized to the mean body size of all individuals present in the population. We used head width (HW) as a body size measurement due to its strong positive correlation with body mass in this species [see also 53]. The scaled mass index for every individual *i* was calculated as follows:

$$\mathop {SMI}\nolimits_{i} = \mathop {BM}\nolimits_{i} \left[ {\frac{{\mathop {HW}\nolimits_{0} }}{{\mathop {HW}\nolimits_{i} }}} \right]^{{\mathop b\nolimits_{SMA} }}$$

where HW_0_ (=21.92 mm) is the arithmetic mean of HW for the population and *b*_*SMA*_ (=3.888) the slope of the standardized major axis (SMA) regression of ln (*BM*) on ln (*HW*). We used the software RMA [54] to calculate the value of *b*_*SMA*_.

#### Assessment of parasitism pattern

Fresh faeces collected opportunistically from handling bags or traps were weighted, directly homogenized with 10% formaldehyde and stored in 2 ml screw cap Sarstedt tubes. Parasite eggs and oocysts were identified under microscopic examination following a slightly modified Ritchie’s ether sedimentation method [55]. Parasites were further classified up to the genus level based on egg or oocyst shape, size and internal structure [56–58], and prevalence, morphotype richness and occurrence of multiple morphotype infections were used to characterize the pattern of parasitism, as detailed in Rakotoniaina et al. [59].

To control for potential observer bias, we used blind observation by coding samples prior to laboratory analysis of hair cortisol levels and faecal parasites.

### Modelling outline and candidate set of models

#### Semi-annual survival

In order to statistically estimate the link between HCC and SMI, as well as semi-annual survival (*Φ*), we used multistate capture-mark-recapture models [60–64] implemented in the program MARK version 8.0 [65], which account for recapture (*p*) and state-transition (*ψ*) probabilities. For each capture session, each individual was first assigned to a high or low HCC and SMI state, using the population median HCC or SMI value of the considered session as a cut-off point. Afterwards, in order to check if the correlation with survival is stronger at high ends of the health indicator values, we explored models where the categorization cut-off was based on the third quartile of HCC and SMI values. We could not use actual HCC and SMI values because modelling individually time-varying covariates (a different covariate value per individual at each recapture event) in MARK requires including a value of the covariates at each capture event even for missing (not recaptured) animals. Therefore, the multistate approach (using HCC/SMI categories) allows us to incorporate a variable value of the covariates between capture events (by accounting for *ψ*) while controlling for missing individuals (by accounting for *p*). Unlike HCC and SMI, the effect of parasitism on survival could not be modelled using this approach since we could not obtain faecal samples for every single individual at every single capture event. Therefore, indices of parasite infection were only considered for the assessment of reproductive season survival (see below).

Following Burnham and Anderson [66], we constructed a priori a candidate set of biologically plausible models (see Additional file 1). We assessed the goodness-of-fit of the global models and obtained an estimation of the variance inflation factor *ĉ* with the median-*ĉ* approach implemented in MARK. This method suggested that our data were slightly overdispersed [models with categorization set using the median: *ĉ(HCC)* = 1.204*, ĉ(SMI)* = 1.432; models with categorization set using the third quartile: *ĉ(HCC)* = 1.213*, ĉ(SMI)* = 1.432], thus model selection statistics were adjusted accordingly. Owing to the rather small sample size, we based our model selection on AICc (or QAICc in the presence of overdispersion) [67], which is an adjusted variant of the Akaike’s information criterion (AIC). The difference (Δ_*i*_) between the AICc of the most parsimonious model and a given model *i* and the normalized Akaike weights (*w*_*i*_) were used to interpret the results of model selection. Hence, models with Δ_*i*_ ≤ 2 were considered to have a strong support while models with 4 < Δ_*i*_ < 7 have intermediate support and models with Δ_*i*_ > 10 have negligible support [66]. The relatively low values of the Akaike weights of our top models (<0.9) for the HCC and the SMI datasets indicated model selection uncertainty and therefore, we adopted multi-model inference techniques over a confidence subset of models (all models with relative likelihood >0.05; see Additional files 2, 3). Thus, the importance of a variable [given as *w*_+_ (variable)] was determined by summing the Akaike weights of models containing the variable of interest. Parameter estimates and their unconditional standard errors were calculated by averaging over all models in our confidence subset of models [66, 68].

We established all our candidate model sets by including factors known to influence mouse lemurs’ survival [38] and consequently considered the factors sex (*s*) and time (*t*) in addition to our measure of the animal condition (*c*; high/low HCC or SMI). As a global model, we used $\varPhi \left( {c*s + t} \right)\;p\left( {s + t} \right) \, \psi \left( {c*t} \right)$ (*: interactive effect, +: additive effect). Subsequently, all possible additive combinations of *c*, *s* and *t* and their single effects were used to model survival probability (*Φ*). Recapture probability (*p*) was additionally considered to be time dependant or constant over time. The condition index (*c*) was not included to model recapture probability since previous studies have reported a lack of a link between stress responses and previous capture experience [69], but also an increasing recapture probability (“trap happiness”) of most individuals in this mouse lemur population [38], suggesting no long-term physiological cost of capture activities. Finally, we further modelled state-transition probability (*ψ*) to depend only on *c.* We fitted a total of 54 models for each condition index by considering all combinations of parameterization used for *Φ, p* and *ψ*.

#### Breeding season survival

We further estimated the potential association between our health indicators (HCC, SMI and parasitism) and survival (*Φ*) and recapture probabilities (*p*) over the breeding season by using the “Cormack-Jolly-Seber” model for open populations [70–72] implemented in MARK. In order to get an accurate estimation of (*Φ*) and (*p*) over the reproductive season, we used data from monthly trapping sessions conducted between the end of the dry season (September 2012) and the end of the rainy season (April 2013). However, as data for January and February 2013 were missing, we controlled for the bias that this gap may have induced to the estimation of (*Φ*) and (*p*) by manually adjusting the time interval of trapping sessions between December and March (3 months instead of one) in our models. Thereafter, we proceeded in two steps. First, we established a starting set of models by using $\varPhi \left( {s* t} \right)\;p\left( {s + t} \right)$ as the global model and further comparing all possible permutation of models with an effect of $\left( {s + t} \right)$, *s* and *t* on survival probability and *s* and *t* on recapture probability along with a constant *Φ* and *p* (see Additional file 4). Then, our health parameter values (HCC, SMI, parasite morphotype richness, overall prevalence and multiple species infection) were successively included as an individual covariate (for the first month only) to the most parsimonious model (basic model) among the starting set to assess if their inclusion improved the fit of the model. Additionally, we checked for potentially normalizing selection that might favour optimal HCC and body mass values and therefore tested for a quadratic effect of HCC and SMI on survival probabilities. We also fitted models including natural log-transformed HCC and SMI values to our monthly capture data. As above, the goodness-of-fit of the global model was assessed using the median-*ĉ* approach (all *ĉ* were <1) and model selection was based on the information theoretical approach [66].

Additionally, we tested for potential intercorrelations among HCC and the other health indicators. If existing, those correlations might mask or interfere with the assessment of their independent links to survival. Yet, no association was detected; neither between HCC and SMI (*r* ^*2*^ = 0.006, *df* = 227, *p* = 0.247) nor between HCC and parasitism pattern (species richness: *r* ^*2*^ = 0.053, *df* = 46, *p* = 0.112; overall prevalence: *r* ^*2*^ = 0.068, *df* = 46, *p* = 0.073; multiple infection: *r* ^*2*^ = 0.062, *df* = 46, *p* = 0.087).

## Results

### Semi-annual survival relative to HCC values

**Fig. 1:**
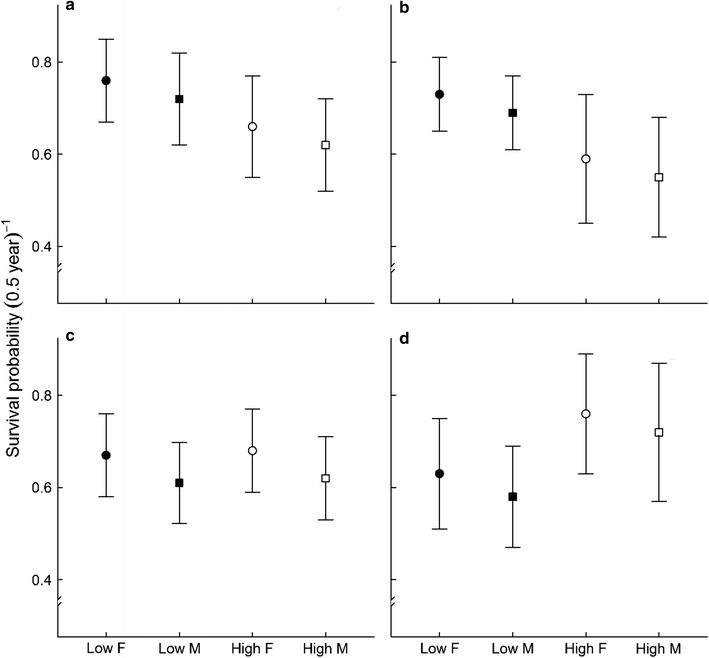
**S**emi-annual survival probabilities of *M. murinus.* Estimates are relative to: hair cortisol concentration (**a** and **b**), where the categorization cut-off is the median (**a**), or the third quartile (**b**); and the scaled mass index (**c** and **d**), where the categorization cut-off is the median (**c**) or the third quartile (**d**). Presented are model-averaged maximum likelihood estimates and unconditional standard errors (*Filled symbols*/*low* low value of the condition index, *Open symbols/high* high value of the condition index, *Circles/F* females, *Squares/M* males). Estimates are averaged (geometric mean) over capture sessions

Multistate models applied on HCC revealed that survival is lower for mouse lemurs with elevated levels of hair cortisol. The gap in survival probability between low and high HCC individuals is larger at high ends of HCC values (Fig. 1a, b). Individuals with low HCC had on average (based on geometric mean across years regardless of sex) a 9.8% higher chance to survive than those with high HCC when the categorization was set using the median HCC value. This gap increased to up to 13.9% when the categories were defined using the third quartile value (Fig. 1a, b). In both cases, in addition to the HCC effect, the best-supported models (Δ_*i*_ < 2) also suggested a sex difference in survival (Table 1). Females survived relatively better than males (Fig. 1a, b; geometric means over time where the median is used as categorization cut-off: *Φ*_low HCC F_ = 0.758, *Φ*_low HCC M_ = 0.724, *Φ*_high HCC F_ = 0.664, *Φ*_high HCC M_ = 0.622; geometric means over time where the third quartile is used as categorization cut-off: *Φ*_low HCC F_ = 0.729, *Φ*_low HCC M_ = 0.694, *Φ*_high HCC F_ = 0.594, *Φ*_high HCC M_ = 0.550). Yet, multi-model inference emphasized that the relative importance of the effect of HCC on survival was higher than the effect of sex independently of the method used to set the categories [median cut-off: *w*_+_ (*HCC*) = 0.701; *w*_+_ (*sex*) = 0.504; third quartile cut-off: *w*_+_ (*HCC*) = 0.756; *w*_+_ (*sex*) = 0.487], further highlighting the strong support for a lowered survival of individuals with high HCC values.

**Table 1:** Model selection statistics (multistate approach) for semi-annual survival (*Φ*), recapture (*p*) and state-transition (*ψ*) probabilities of *M. murinus* depending on hair cortisol concentration and general body condition (measured as scaled mass index)

Rank	Model	K	QDEV	QAICc	Δ_*i*_	*w*_*i*_
Hair cortisol concentration (categorization cut-off: median)						
1	$\varPhi \left( c \right)\;p\left( t \right) \, \psi \left( c \right)$	8	200.78	515.10	0	0.147
2	$\varPhi \left( s \right)\;p\left( t \right) \, \psi \left( c \right)$	8	201.90	516.23	1.13	0.142
3	$\varPhi \left( {c + s} \right) \, p\left( t \right) \, \psi \left( c \right)$	9	199.96	516.44	1.34	0.126
4	$\varPhi \left( c \right) \, p\left( \cdot \right) \, \psi \left( c \right)$	5	208.91	516.88	1.77	0.120
*53*	$\varPhi \left( {c*s + t} \right) \, p\left( {s + t} \right) \, \psi \left( {c*t} \right)$	*20*	*192.77*	*534.29*	*19.19*	*0.0001*
Hair cortisol concentration (categorization cut-off: third quartile)						
1	$\varPhi \left( c \right) \, p\left( t \right) \, \psi \left( c \right)$	8	146.94	465.86	0	0.230
2	$\varPhi \left( {c + s} \right) \, p\left( t \right) \, \psi \left( c \right)$	9	146.08	467.16	1.30	0.120
3	$\varPhi \left( c \right) \, p\left( \cdot \right) \, \psi \left( c \right)$	5	154.75	467.31	1.45	0.111
4	$\varPhi \left( s \right) \, p\left( t \right) \, \psi \left( c \right)$	8	148.66	467.59	1.73	0.097
*54*	$\varPhi \left( {c*s + t} \right) \, p\left( {s + t} \right) \, \psi \left( {c*t} \right)$	*20*	*138.21*	*484.33*	*18.47*	*0.00002*
Scaled mass index (categorization cut-off: median)						
1	$\varPhi \left( s \right) \, p\left( t \right) \, \psi \left( c \right)$	7	114.35	330.82	0	0.272
2	$\varPhi \left( c \right) \, p\left( t \right) \, \psi \left( c \right)$	7	115.43	331.90	1.08	0.158
*54*	$\varPhi \left( {c*s + t} \right) \, p\left( {s + t} \right) \, \psi \left( {c*t} \right)$	*16*	*109.73*	*346.67*	*15.85*	*0.0004*
Scaled mass index (categorization cut-off: third quartile)						
1	$\varPhi \left( c \right) \, p\left( t \right) \, \psi \left( c \right)$	7	102.53	323.87	0	0.229
2	$\varPhi \left( {c + s} \right) \, p\left( t \right) \, \psi \left( c \right)$	8	101.34	324.85	0.99	0.140
3	$\varPhi \left( s \right) \, p\left( t \right) \, \psi \left( c \right)$	7	103.72	325.06	1.19	0.127
4	$\varPhi \left( c \right) \, p\left( {s + t} \right) \, \psi \left( c \right)$	8	102.03	325.55	1.68	0.099
*54*	$\varPhi \left( {c*s + t} \right) \, p\left( {s + t} \right) \, \psi \left( {c*t} \right)$	*16*	*96.91*	*338.72*	*14.85*	*0.0001*

Only models with Δ_*i*_ ≤ 2 and the global model (in italic) are shown here with the number of parameters (K), the quasi-likelihood adjusted deviance (QDEV), the quasi-likelihood adjusted AIC for small sample size (QAICc), the difference between the QAICc of the top model and a given model *i (*Δ_*i*_ *)* and the Akaike weights (*w*_*i*_). Variables considered are the condition index (*c,* which can indicate HCC or SMI values), sex (*s*) and time (*t*). Constant parameters are noted (.). Interactions are indicated by (*) and additive effects by (+)

In both approaches, our candidate set of models showed limited support for between-season variation in survival [median cut-off: *w*_+_ (*t*) = 0.170; third quartile cut-off: *w*_+_ (*t*) = 0.108) but instead a strong variability of recapture probabilities through time [median cut-off: *w*_+_ (*t*) = 0.805; third quartile cut-off: *w*_+_ (*t*) = 0.756]. All models in the confidence sets supported that a transition to a given state (high or low HCC) depended mainly on the current state of the individual (Table 1).

### Semi-annual survival relative to SMI values

We found only weak support for an effect of SMI on survival in comparison to the effect of sex when categories were established according to the median SMI value [*w*_+_ (*sex*) = 0.625; *w*_+_ (*SMI*) = 0.441]. On average (based on the geometric mean across seasons), females had around 6% higher chance to survive to the next season than males but the difference of survival probability between conditions was negligible (Fig. 1c). However, our results also suggested that *M. murinus* in very good body condition (categories based on the third quartile regardless of sexes) survive on average 13.7% better than low condition animals (Fig. 1d), and strong support for a positive effect of SMI on survival was obtained with those models [*w*_+_ (*SMI*) = 0.744; *w*_+_ (*sex*) = 0.513].

Additionally, a time varying recapture probability structure was strongly supported by our confidence set of models in both scenarios [median cut-off: *w*_+_ (*t*) = 0.895; third quartile cut-off: *w*_+_ (*t*) = 0.884].

### Health indicators and survival over the breeding season

**Fig. 2:**
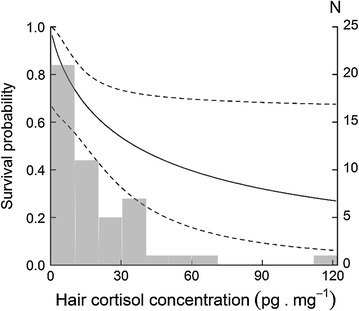
Monthly survival probabilities of *M. murinus* relative to the hair cortisol concentration. Considered is linear effect on the natural log-transformed HCC (values presented here are back transformed to the original scale). Presented are main effect (*solid line*) and 95% CI (*dashed lines*). Histograms represent the sample size for each category

The most parsimonious model (basic model) among the starting set contained time varying survival and recapture probabilities $\left( {\varPhi \left( t \right)\;p\left( t \right)} \right)$. All models including HCC as a predictor of survival had a better fit than the basic model (Table 2). The top model (with natural log-transformed HCC) was more than four times better supported than the basic model [*w* (ln(*HCC*)) = 0.366, *w* (*t*) = 0.0.83; 0.366/0.083 = 4.404]. In the two best-supported models (Δ_*i*_ < 2) we found a negative relationship between HCC and survival [ln (HCC), Fig. 2a; HCC, Fig. 2b]. In contrast, we found no evidence of a link between survival and SMI or parasitemia, as the basic model performed better than all the models containing the other health indicators as covariates (SMI, multiple parasite species richness, parasite morphotype richness and overall prevalence; Table 2).

**Table 2:** Model selection statistics for monthly survival (*Φ*) and recapture (*p*) probabilities of *M. murinus*

Rank	Model	K	DEV	AICc	Δ_*i*_	*w*_*i*_
1	$\varPhi \left( {t + ln\left( {HCC} \right)} \right) \, p\left( t \right)$	10	193.11	215.04	0	0.366
2	$\varPhi \left( {t + HCC} \right) \, p\left( t \right)$	10	193.83	215.76	0.72	0.256
3	$\varPhi \left( {t + HCC + HCC^{2} } \right) \, p\left( t \right)$	11	193.65	217.98	2.94	0.084
*4*	$\varPhi \left( t \right) \, p\left( t \right)$	*9*	*198.44*	*218.01*	*2.97*	*0.083*
5	$\varPhi \left( {t + \,mult} \right) \, p\left( t \right)$	10	196.51	218.44	3.40	0.067
6	$\varPhi \left( {t + \,rich} \right) \, p\left( t \right)$	10	197.70	219.63	4.59	0.037
7	$\varPhi \left( {t + \,ln\left( {SMI} \right)} \right) \, p\left( t \right)$	10	197.84	219.77	4.73	0.034
8	$\varPhi \left( {t + SMI} \right) \, p\left( t \right)$	10	197.95	219.88	4.84	0.033
9	$\varPhi \left( {t + prev} \right) \, p\left( t \right)$	10	198.41	220.34	5.30	0.026
10	$\varPhi \left( {t + SMI + SMI^{2} } \right) \, p\left( t \right)$	11	197.15	221.49	6.45	0.015

In this Cormack-Jolly-Seber approach, monthly survival (*Φ*) and recapture (*p*) probabilities are assessed depending on hair cortisol concentration (*HCC*), general body condition (measured as scaled mass index, *SMI*) and patterns of parasitism (multiple species infection: *mult;* parasite morphotype richness: *rich;* overall prevalence: *prev*). The basic model (in italic) and models containing covariates are shown with the number of parameters (K), the deviance (DEV), the adjusted AIC for small sample size (AICc), the difference between the AICc of the top model and a given model *i (*Δ_*i*_ *)* and the Akaike weights (*w*_*i*_)

## Discussion

In this study, we tested the hypothesis that high HCC (as a measure of long-term activation of the HPA axis) translates into reduced survival. Using wild mouse lemurs previously known to face condition-dependent mortality [37], we further tested the correlation of two other health indicators (body condition and patterns of parasitism) with survival in order to compare their predictive power for fitness outcomes. As predicted by the Cort-fitness hypothesis [16], both semi-annual survival and survival over the reproductive period were negatively associated with the level of accumulated hair cortisol. The first approach revealed that the relationship between HCC and survival is particularly strong at the high end of HCC values. Furthermore, our result suggested that individuals in extremely good condition enjoy higher survival probabilities than the ones with mid to low SMI values. In contrast, there was little support for the effects of SMI and measures of parasitism (multiple parasite species richness, parasite morphotype richness and overall prevalence) on survival over the reproductive period.

### The relationship between stress and survival in a wild primate population

Our results provide general support for the Cort-fitness hypothesis which posits that elevated GCs are associated with a decline in one component of fitness [16]. Under this hypothesis, individuals in poor quality are assumed to perceive their environment as challenging and therefore secrete higher levels of GC than good quality individuals. However, similar to what has been reported by several other studies [73, 74], our data suggest that the range of GC concentration can be wide and thus, emphasize the need for caution when interpreting differences in GC concentrations without a proper assessment of their biological significance [75]. Individual significant differences in GC concentrations do not necessarily translate into significant diverging biological effects and conversely, slight changes in hormonal levels could be important. For instance, Pride [8] found that GC can be a sensitive indicator of survival probabilities but especially at very high values. Although natural selection can strongly operate on GC regulation, the high inter-individual variability for this trait seen in the wild could be maintained if the divergent stress responses offer alternative strategies with differing payoffs depending on environmental conditions [76, 77].

Furthermore, the assessment of how HCC correlates with survival of *M. murinus* over the reproductive season supported the notion that the relationship between GC and fitness can be context-dependent [9, 78]. Indeed, the benefit of having a relatively low stress load seems to be maximized prior to entering the breeding season as previously observed for various bird species [17, 79]. Individuals that are already strongly affected by challenging conditions during the dry season might not be able to cope with the additional costs related to the mating season. This brief period is particularly challenging for male mouse lemurs, which show a drastic increase in mortality coupled with significant body condition deterioration over the mating season [38, 46] that might be proximately mediated by the adverse physiological consequences of the stress accumulated over the dry season. Unlike females, which hibernate for several months, male mouse lemurs stay active and only undergo daily torpor during the dry winter [46, 80]. Staying active and the need to be energetically prepared to face the mating season [81] seems to physiologically affect males, which showed a higher HCC than females at the end of the dry season [59]. However, we could not detect this sex difference in the present data set, probably due to the limited sample size. This limitation emphasizes the importance of chronologically isolating specific processes in the life cycle in order to comprehend the proximate mechanisms that impact the survival of a given population.

High mortality rate as a cost of high reproductive success was described for several study systems [82, 83], and GC hormones were suggested to be central in mediating this trade-off [84]. Lee and Cockburn [85] proposed that during the mating period, animals (especially males) may exhibit an adaptive stress response, which can compromise their survival but promotes reproductive fitness by permitting a redirection of energy to reproduction. For instance, such terminal investment was detected in male arctic ground squirrels [86], and it was evoked that it may occur in mammalian species with similar life history traits characterized by a single annual breeding opportunity per year coupled with high between-year mortality [84]. This physiological adaptation might occur in male grey mouse lemurs facing strong intrasexual competition over access to receptive females [45] and thus, it could severely affect individuals that are showing already signs of high stress load at the beginning of the mating season. An estimation of the reproductive success of animals showing high GC levels will help to test this hypothesis.

Several reasons could lead to the high mortality of chronically stressed individuals, including impaired immune and inflammatory responses leading to impaired resistance to diseases [87], or a maladaptive adrenocortical response to additional unpredictable stressors that might impair the animals’ coping ability [88]. However, our results seem to argue against the hypothesis of increased mortality due to increased parasitism. All models that included an indicator of parasitism failed to support a monthly survival trend and suggested that multiple parasite species infection, parasite morphotype richness, and overall prevalence were poor predictors of individual survival. Acquired immunity against helminth infections [41] might further explain the relatively low selective pressure on parasitism. As parasite virulence and host tolerance might also be highly variable, these findings highlight the degree of uncertainty associated with the use of basic measures of parasitism as biomarker of health without information on parasite pathogenicity.

Additionally, chronically stressed individuals could fail to mount an adaptive HPA activity response to an acute stressor such as predation which might increase their vulnerability during the mating season. For instance, the grey mouse lemur is known to be preyed upon by several predators such as snakes, owls or another lemur (*Mirza coquereli*), and although they face a continuously high predation risk [42, 89, 90], this threat might be maximal at the peak of the activity period of both predators and prey. In this case, an inappropriate physiological response to the presence of a predator could be translated into reduced reaction time or escaping ability of the high-stress individuals. Several studies have reported that GC responses to acute stressors were down-regulated in animals exposed to chronic stress [3, 91]. For instance, in lemurs, Tecot [92] found that *Eulemur rubriventer* showed an attenuated GC response to known seasonal environmental challenges in altered habitats. While this response could be aimed at reducing the detrimental effects of chronic elevation of GC levels, it may negatively affect the capacity of an animal to face acute life-threatening stressors.

Age is a factor that could influence the GC-survival relationship, but it could unfortunately not be addressed in this study. Previous studies suggested that both survival and GC profile are age-dependent in grey mouse lemurs. For instance, Kraus et al. [38] reported lower survival of juveniles over the dry winter but no significant difference between juvenile and adult survival probabilities over the summer. Also, while older individuals were found to have higher faecal GC metabolites during the breeding season [40], Rakotoniaina et al. [59] showed that HCC was higher in juvenile *M. murinus*. The contradicting results found in those studies might have arisen from the different matrices used to assess physiological stress but also from the definition of age: while the first study used estimates of individual age, the latter applied age categorization. This age-GC link was also detected in various study systems [93–97] and is mainly assumed to be associated with the impaired ability of aged individuals to cope with challenges [98]. Considering age is therefore recommended in future studies examining the link between GC and fitness.

### Body condition, stress and survival

As suggested previously [37], we found that mortality is condition-dependent in *M. murinus*. In fact, very good condition (measured as scaled mass index) was associated with a high semi-annual survival probability. Poor quality individuals can be more vulnerable to diseases [99] and predation [100] but also have a lower capacity to face competition [101]. While body mass has been reported to consistently decline in chronically stressed individuals [102], it is still necessary to disentangle and define the causal effect of body condition and stress hormones on the survival output at high values of these health indicators. As body condition and GC levels were not correlated in our study population, selection on either of these traits should not influence the detection of survival selection on the other one [74].

Also, body condition was not correlated with monthly survival, which may indicate that different selective pressures acting on this trait might have opposite effects during the mating season. For instance, if individuals in good condition are more active than weak individuals in this period, this may increase their probability to encounter predators [103–105]. The benefits of being in better condition (e.g. low susceptibility to disease, high success in resource competition) could therefore be curtailed by increased predation risk. Yet, if body condition does not correlate with survival from low to mid values, as suggested by the multistate analysis, this failure of body condition to explain monthly survival could also arise from the limited sample size being used, which does not allow us to detect this trend. Overall, our results indicate the possibility that physiological traits are under stronger selection in terms of survival consequences than body condition. Additional studies of the heritability and effects of these health indicators on reproductive success would be needed, however, to confirm the overall selective potential for these traits.

### Hair cortisol concentration as a reliable health indicator

The gold standard of validating a biological indicator of health is to show that it correlates with fitness. Here, we demonstrate for the first time that HCC exhibited such a correlation in a wild population. Although the exact mechanism of incorporation of cortisol in the hair shaft is not yet well understood [106–108], it is mostly assumed that cortisol contained in hair is representative of free systemic concentration [For a review see 21, 22 but see 109, 110]. Similar to our study, Patterson et al. [74] found that free GC hormone levels may be more relevant than morphological traits as a predictor of survival in white-crowned sparrows.

The fundamental advantage of using hair as a matrix to assess cortisol levels is the broadness of the time window reflected by HCC. While HCC is assumed to account for up to several months of stress load, traditionally used biomarkers of stress (blood, faeces, urine, saliva) are point estimators and could fail to describe the true individual condition. Under natural conditions where animals cannot be continuously observed and sampled, it is difficult to obtain a reliable measure of the baseline GC level with such biomarkers. However, most studies that investigated this aspect in the wild rely solely on limited sampling of indicators of short-term stress responses that are likely affected, for instance, by daily level fluctuations or individual stressful events experienced prior to sampling [e.g. 73, 111]. Overall, this might explain the large inconsistency in the results so far reported from studies that attempted to link stress and fitness [16]. At present, the lack of precise information on hair growth rate in *M. murinus* limits our estimation about the period of accumulation of cortisol recorded with the hair samples. However, when an animal was captured and sampled early in September, we observed that the hair had fully regrown after a subsequent recapture of the same individual in December. Thus, we are confident that HCC in our study reflected the mean cortisol load over, at least, a substantial part of each period between sampling sessions.

Moreover, HCC has been shown to be heritable and reported to represent an individual trait that is affected by genes and environment [31]. Since cortisol is known to be closely linked to a series of other phenotypic traits [76, 112], levels of hair cortisol may indicate individual quality, where low quality individuals that might perceive their environment as more challenging secrete higher cortisol levels [113]. For instance in our study population, considering that HCC reflects an accumulation of cortisol over an extended period of time, and that individuals are assumed to face similar extrinsic pressures, it is very likely that individual differences in HCC reflect true differences in condition rather than a potential difference in exposure to various stressors. However, consistent individual behavioural responses to external stimuli, also refered to as “personality” [sensu 114], could interfere with this individual quality-GC profile relationship. Indeed, several studies have demonstrated that specific personality traits such as boldness can strongly correlate with HPA axis responses [115–118]. An investigation of such a relationship by conducting personality tests combined with HCC measurements might enrich the interpretation of information obtained from hair cortisol levels.

## Conclusions

This study provides support for the Cort-fitness hypothesis by demonstrating that survival is negatively associated with levels of hair cortisol concentrations in a wild grey mouse lemur population. This study therefore provides a first confirmation of the predictive power of HCC variation on individual fitness in a wild setting. Moreover, we demonstrate that, while GC, body condition and parasite resistance could all influence individual survival, their effects might differ in strength. Thus, we emphasize that care must be taken when interpreting such indices without prior knowledge of their effect on fitness. Although our approach is correlational and does not permit the identification of the exact causes of mortality, it suggests that variation in GC hormone concentrations alone may underlie demographic fluctuations of natural populations. Thus, these results highlight the need to consider environmental pressures that can affect GC levels as potential threats to survival. Since population decline is often hard to measure, the assessment of an individual health indicator such as long-term stress levels could, therefore, provide an easier alternative for detecting issues emerging at the population level and ultimately predicting wild populations’ responses to environmental challenges.

## Additional files

**Additional file 1.** Full set of starting candidate models. This additional file presents the full list of candidate set of biologically plausible models that were constructed *a priori* and used for the semi-annual survival estimation.

**Additional file 2.** Confidence set of models for semi-annual survival of *M. murinus* depending on hair cortisol concentration. This additional file presents the full list of models with relative likelihood > 0.05 obtained from the semi-annual survival estimation of *M. murinus* depending on hair cortisol concentration.

**Additional file 3.** Confidence set of models for semi-annual survival of *M. murinus* depending on scaled mass index. This additional file presents the full list of models with relative likelihood > 0.05 obtained from the semi-annual survival estimation of *M. murinus* depending on scaled mass index.

**Additional file 4.** Starting set of models for monthly survival assessment of *M. murinus*. This additional file presents the full list of starting set of models used in order to identify the basic model for the monthly survival estimation of *M. murinus* depending on hair cortisol concentration and scaled mass index.

## Abbreviations

GC: glucocorticoid
HCC: hair cortisol concentration
HPA axis: hypothalamic–pituitary–adrenal axis
BM: body mass
SMI: scaled mass index
