# Supplementary material for: Hair cortisol concentrations correlate negatively with survival in a wild primate population
Source: BMC Ecol. 2017 Sep 1;17:30. doi: 10.1186/s12898-017-0140-1 (PMC5579956; doi:10.1186/s12898-017-0140-1)
Supplement: Supplementary file 1 — Additional file 1. Full set of starting candidate models. This additional file presents the full list of candidate set of biologically plausible models that were constructed a priori and used for the semi-annual survival estimation. [file 12898_2017_140_MOESM1_ESM.pdf]

Full set of starting candidate models.

We considered the following factors: condition index (*c*, which can indicate HCC or SMI values), sex (*s*), and time (*t*). Constant parameters are noted (.). Interactions are indicated by (*) and additive effects by (+). All possible combinations of parameters given below were modelled and tested. Global models are in bold.

Survival probability *Φ*	Recapture probability *p*	State-transition probability *ψ*
Semi-annual survival (Multistate models)		
c * s + t	***s + t***	***c * t***
c + s + t	*t*	*c*
*c * s*	(.)	
c + s		
c + t		
s + t		
c		
s		
t		
Monthly survival (Cormack-Jolly-Seber models where explanatory variables were later added as an individual covariates)		
s * t	***s + t***	
s + t	*s*	
s	*t*	
t	(.)	
(.)		
