# Supplementary material for: Hair cortisol concentrations correlate negatively with survival in a wild primate population
Source: BMC Ecol. 2017 Sep 1;17:30. doi: 10.1186/s12898-017-0140-1 (PMC5579956; doi:10.1186/s12898-017-0140-1)
Supplement: Supplementary file 2 — Additional file 2. Confidence set of models for semi-annual survival of M. murinus depending on hair cortisol concentration. This additional file presents the full list of models with relative likelihood > 0.05 obtained from the semi-annual survival estimation of M. murinus depending on hair cortisol concentration. [file 12898_2017_140_MOESM2_ESM.pdf]

Confidence set of models (all models with relative likelihood > 0.05) for semi-annual survival of *M. murinus* depending on hair cortisol concentration.

Presented are the number of parameters (K), the deviance (QDEV), the quasi-likelihood adjusted second order AIC (QAICc), the difference between the QAICc of the top model and a given model *i* (Δ_i) and the Akaike weights (w_i).

Rank	Model	K	QDEV	QAICc	Δ_i	w_i
Categorization cut-off: median						
1	$\Phi(c)\ p(t)\ \psi(c)$	8	200.78	515.10	0	0.208
2	$\Phi(s)\ p(t)\ \psi(c)$	8	201.90	516.23	1.13	0.119
3	$\Phi(c + s)\ p(t)\ \psi(c)$	9	199.96	516.44	1.34	0.107
4	$\Phi(c)\ p(.)\ \psi(c)$	5	208.91	516.88	1.77	0.086
5	$\Phi(c)\ p(s + t)\ \psi(c)$	9	200.73	517.21	2.11	0.072
6	$\Phi(s)\ p(.)\ \psi(c)$	5	210.01	517.98	2.87	0.050
7	$\Phi(t)\ p(t)\ \psi(c)$	10	199.40	518.06	2.96	0.048
8	$\Phi(c + t)\ p(t)\ \psi(c)$	11	197.29	518.15	3.05	0.045
9	$\Phi(c + s)\ p(.)\ \psi(c)$	6	208.16	518.23	3.12	0.044
10	$\Phi(s)\ p(s + t)\ \psi(c)$	9	201.84	518.32	3.22	0.042
11	$\Phi(c + s)\ p(s + t)\ \psi(c)$	10	199.86	518.52	3.42	0.038
12	$\Phi(c * s)\ p(t)\ \psi(c)$	10	199.88	518.54	3.44	0.037
13	$\Phi(s + t)\ p(t)\ \psi(c)$	11	198.81	519.67	4.57	0.021
14	$\Phi(t)\ p(s + t)\ \psi(c)$	11	198.96	519.81	4.71	0.020
15	$\Phi(c + s + t)\ p(t)\ \psi(c)$	12	196.76	519.84	4.74	0.020
16	$\Phi(c + t)\ p(s + t)\ \psi(c)$	12	197.09	520.16	5.06	0.017
17	$\Phi(c * s)\ p(.)\ \psi(c)$	7	208.10	520.29	5.19	0.016
18	$\Phi(c * s)\ p(s + t)\ \psi(c)$	11	199.80	520.66	5.56	0.013
Categorization cut-off: third quartile						
1	$\Phi(c)\ p(t)\ \psi(c)$	8	146.94	465.86	0	0.230
2	$\Phi(c + s)\ p(t)\ \psi(c)$	9	146.08	467.16	1.30	0.120
3	$\Phi(c)\ p(.)\ \psi(c)$	5	154.75	467.31	1.45	0.111
4	$\Phi(s)\ p(t)\ \psi(c)$	8	148.66	467.59	1.73	0.097
5	$\Phi(c)\ p(s + t)\ \psi(c)$	9	146.88	467.97	2.11	0.080
6	$\Phi(c + s)\ p(.)\ \psi(c)$	6	153.97	468.63	2.77	0.058
7	$\Phi(c + s)\ p(s + t)\ \psi(c)$	10	145.98	469.24	3.38	0.042
8	$\Phi(c * s)\ p(t)\ \psi(c)$	10	146.00	469.26	3.40	0.042
9	$\Phi(s)\ p(.)\ \psi(c)$	5	156.72	469.28	3.42	0.042
10	$\Phi(t)\ p(t)\ \psi(c)$	10	146.17	469.44	3.57	0.038
11	$\Phi(s)\ p(s + t)\ \psi(c)$	9	148.60	469.69	3.82	0.034
12	$\Phi(c + t)\ p(t)\ \psi(c)$	11	144.88	470.34	4.47	0.025
13	$\Phi(c * s)\ p(.)\ \psi(c)$	7	153.89	470.68	4.81	0.021
14	$\Phi(s + t)\ p(t)\ \psi(c)$	11	145.60	471.06	5.19	0.017
15	$\Phi(t)\ p(s + t)\ \psi(c)$	11	145.74	471.20	5.33	0.016
16	$\Phi(c * s)\ p(s + t)\ \psi(c)$	11	145.91	471.37	5.51	0.015
17	$\Phi(s + t)\ p(.)\ \psi(c)$	8	152.78	471.71	5.84	0.012
