# Supplementary material for: Hair cortisol concentrations correlate negatively with survival in a wild primate population
Source: BMC Ecol. 2017 Sep 1;17:30. doi: 10.1186/s12898-017-0140-1 (PMC5579956; doi:10.1186/s12898-017-0140-1)
Supplement: Supplementary file 3 — Additional file 3. Confidence set of models for semi-annual survival of M. murinus depending on scaled mass index. This additional file presents the full list of models with relative likelihood > 0.05 obtained from the semi-annual survival estimation of M. murinus depending on scaled mass index. [file 12898_2017_140_MOESM3_ESM.pdf]

Confidence set of models (all models with relative likelihood > 0.05) for semi-annual survival of *M. murinus* depending on scaled mass index.

Presented are the number of parameters (K), the deviance (QDEV), the quasi-likelihood adjusted second order AIC (QAICc), the difference between the QAICc of the top model and a given model *i* (Δ_i) and the Akaike weights (w_i).

Rank	Model	K	QDEV	QAICc	Δ_i	w_i
Categorization cut-off: median						
1	$\Phi(s)\ p(t)\ \psi(c)$	7	114.35	330.82	0	0.272
2	$\Phi(c)\ p(t)\ \psi(c)$	7	115.44	331.90	1.09	0.158
3	$\Phi(s)\ p(s + t)\ \psi(c)$	8	114.31	332.96	2.14	0.093
4	$\Phi(c + s)\ p(t)\ \psi(c)$	8	114.35	332.99	2.18	0.092
5	$\Phi(c)\ p(s + t)\ \psi(c)$	8	115.01	333.65	2.84	0.066
6	$\Phi(t)\ p(t)\ \psi(c)$	8	115.36	334.01	3.19	0.055
7	$\Phi(c * s)\ p(t)\ \psi(c)$	9	113.72	334.56	3.75	0.042
8	$\Phi(t)\ p(.)\ \psi(c)$	6	120.63	334.94	4.12	0.035
9	$\Phi(s)\ p(.)\ \psi(c)$	5	122.92	335.10	4.29	0.032
10	$\Phi(s + t)\ p(t)\ \psi(c)$	9	114.28	335.13	4.31	0.031
11	$\Phi(c + s)\ p(s + t)\ \psi(c)$	9	114.31	335.15	4.34	0.031
12	$\Phi(t)\ p(s + t)\ \psi(c)$	9	114.98	335.83	5.01	0.022
13	$\Phi(c)\ p(.)\ \psi(c)$	5	123.82	336.00	5.18	0.020
14	$\Phi(c + t)\ p(t)\ \psi(c)$	9	115.29	336.14	5.32	0.019
15	$\Phi(s + t)\ p(.)\ \psi(c)$	7	119.72	336.19	5.37	0.019
16	$\Phi(c * s)\ p(s + t)\ \psi(c)$	10	113.67	336.74	5.92	0.014
Categorization cut-off: third quartile						
1	$\Phi(c)\ p(t)\ \psi(c)$	7	102.53	323.87	0	0.229
2	$\Phi(c + s)\ p(t)\ \psi(c)$	8	101.34	324.85	0.99	0.140
3	$\Phi(s)\ p(t)\ \psi(c)$	7	103.72	325.06	1.19	0.127
4	$\Phi(c)\ p(s + t)\ \psi(c)$	8	102.03	325.55	1.68	0.099
5	$\Phi(c + s)\ p(s + t)\ \psi(c)$	9	101.28	327.00	3.13	0.048
6	$\Phi(c * s)\ p(t)\ \psi(c)$	9	101.34	327.05	3.19	0.047
7	$\Phi(s)\ p(s + t)\ \psi(c)$	8	103.68	327.20	3.33	0.043
8	$\Phi(c)\ p(.)\ \psi(c)$	5	110.64	327.69	3.83	0.034
9	$\Phi(c + t)\ p(t)\ \psi(c)$	9	102.51	328.22	4.36	0.026
10	$\Phi(t)\ p(t)\ \psi(c)$	8	104.73	328.24	4.38	0.026
11	$\Phi(c)\ p(t)\ \psi(c * t)$	11	98.10	328.28	4.42	0.025
12	$\Phi(c + t)\ p(.)\ \psi(c)$	7	107.43	328.77	4.90	0.020
13	$\Phi(c + s)\ p(.)\ \psi(c)$	6	109.63	328.81	4.95	0.019
14	$\Phi(t)\ p(.)\ \psi(c)$	6	110.00	329.18	5.31	0.016
15	$\Phi(c * s)\ p(s + t)\ \psi(c)$	10	101.28	329.22	5.36	0.016

continued

Rank	Model	K	QDEV	QAICc	Δ_i	w_i
Categorization cut-off: third quartile						
16	$\Phi(c + s + t)\ p(t)\ \psi(c)$	10	101.33	329.27	5.40	0.015
17	$\Phi(s)\ p(.)\ \psi(c)$	5	112.29	329.34	5.48	0.015
18	$\Phi(s)\ p(t)\ \psi(c * t)$	11	99.16	329.35	5.48	0.015
19	$\Phi(s + t)\ p(t)\ \psi(c)$	9	103.65	329.37	5.50	0.015
20	$\Phi(c + t)\ p(t)\ \psi(c * t)$	12	97.01	329.47	5.60	0.014
21	$\Phi(c + s + t)\ p(.)\ \psi(c)$	8	106.24	329.76	5.89	0.012
