# Supplementary material for: Hair cortisol concentrations correlate negatively with survival in a wild primate population
Source: BMC Ecol. 2017 Sep 1;17:30. doi: 10.1186/s12898-017-0140-1 (PMC5579956; doi:10.1186/s12898-017-0140-1)
Supplement: Supplementary file 4 — Additional file 4. Starting set of models for monthly survival assessment of M. murinus. This additional file presents the full list of starting set of models used in order to identify the basic model for the monthly survival estimation of M. murinus depending on hair cortisol concentration and scaled mass index. [file 12898_2017_140_MOESM4_ESM.pdf]

Starting set of models for monthly survival assessment of *M. murinus*.

Presented are the number of parameters (K), the deviance (DEV), the second order AIC (AICc), the difference between the AICc of the top model and a given model i (Δ_i) and the Akaike weights (w_i) for the total set including the global model (in bold).

Rank	Model	K	DEV	AICc	Δ_i	w_i
1	$\Phi(t)\ p(t)$	9	16.06	218.01	0	0.562
2	$\Phi(t)\ p(s + t)$	10	15.89	220.20	2.19	0.188
3	$\Phi(s + t)\ p(t)$	10	15.97	220.28	2.28	0.180
4	$\Phi(s + t)\ p(s + t)$	11	15.89	222.60	4.60	0.056
5	$\Phi(.)\ p(t)$	6	32.52	227.61	9.60	0.005
6	$\Phi(s * t)\ p(t)$	14	13.51	227.71	9.70	0.004
7	$\Phi(s)\ p(t)$	7	32.45	229.78	11.78	0.002
8	$\Phi(.)\ p(s + t)$	7	32.51	229.84	11.84	0.002
**9**	$\boldsymbol{\Phi(s * t)\ p(s + t)*}$	**15**	**13.49**	**230.27**	**12.27**	**0.001**
10	$\Phi(s)\ p(s + t)$	8	32.44	232.06	14.05	0.001
11	$\Phi(t)\ p(.)$	4	51.38	242.09	24.09	0.000
12	$\Phi(t)\ p(s)$	7	51.22	248.55	30.55	0.000
13	$\Phi(s + t)\ p(.)$	7	51.38	248.72	30.71	0.000
14	$\Phi(s + t)\ p(s)$	8	51.21	250.83	32.83	0.000
15	$\Phi(s * t)\ p(.)$	11	51.26	257.98	39.97	0.000
16	$\Phi(s * t)\ p(s)$	12	51.12	260.29	42.28	0.000
17	$\Phi(.)\ p(.)$	2	83.32	269.79	51.79	0.000
18	$\Phi(.)\ p(s)$	3	83.19	271.77	53.77	0.000
19	$\Phi(s)\ p(.)$	3	83.32	271.89	53.89	0.000
20	$\Phi(s)\ p(s)$	4	83.19	273.90	55.90	0.000
